# Comparative evaluation of culture systems for shoot propagation and phytochemical characteristics in pokeweed (*Phytolacca americana* L.)

**DOI:** 10.7717/peerj.21812

**Published:** 2026-10-05

**Authors:** Worasitikulya Taratima, Wipa Yaowachai, Attachai Trunjaruen, Pitakpong Maneerattanarungroj, Prathan Luecha

**Affiliations:** 1 Department of Biology, Faculty of Science, Khon Kaen University, Khon Kaen, Thailand; 2 Queen Sirikit Botanic Garden, Botanical Garden Organization, Chiang Mai, Thailand; 3 Department of Biology, Faculty of Science, Burapha University, Chon Buri, Thailand; 4 Faculty of Veterinary Medicine, Khon Kaen University, Khon Kaen, Thailand; 5 Department of Pharmacognosy and Toxicology, Faculty of Pharmaceutical Science, Khon Kaen University, Khon Kaen, Thailand

**Keywords:** Pokeweed, Temporary immersion system, Organogenesis, Phenolics, Antioxidant activity, Bioreactor

## Abstract

**Background:**

*Phytolacca americana* L. (pokeweed) is a medicinal plant containing diverse bioactive compounds, including phenolics, flavonoids, and saponins. Variation in phytochemical accumulation and concerns associated with harvesting from natural populations highlight the need for controlled and sustainable propagation systems. This study compared solid, liquid, and temporary immersion system (TIS) cultures of *P. americana* nodal explants containing axillary buds and evaluated their effects on plant growth and phytochemical characteristics.

**Methods:**

Nodal explants were cultured on solid and liquid media or in a TIS bioreactor. Six TIS regimes combining 5 or 10 min immersion durations with 1, 2, or 3 immersions per day were evaluated. Growth, callus formation, biomass, total phenolic content (TPC), total flavonoid content (TFC), and 2,2-diphenyl-1-picrylhydrazyl (DPPH) radical-scavenging activity were determined. Multivariate analyses were used to characterize treatment responses.

**Results:**

All treatments showed 100% explant survival and response. Liquid culture produced the weakest shoot and leaf development, whereas TIS generally supported greater shoot growth. T6 (10 min, three immersions/day) produced the highest shoot length (5.69 ± 0.83 cm), leaf length (4.79 ± 0.55 cm), and dry weight (0.12 ± 0.03 g). T5 produced the highest TPC (14.82 ± 0.10 mg gallic acid equivalents per gram (GAE/g) extract), solid culture the highest TFC (31.25 ± 0.94 mg quercetin equivalents per gram (QE/g) extract), and T1 the strongest DPPH radical-scavenging activity (IC_50_ = 553.25 ± 16.46 µg/mL). Multivariate analyses revealed distinct response patterns among culture systems and identified key traits contributing to treatment discrimination.

**Conclusion:**

Culture system and immersion regime differentially influenced the growth and phytochemical characteristics of *P. americana* nodal explants. TIS, particularly T4–T6, showed potential for enhancing shoot development and biomass accumulation, providing a basis for further optimization of *P. americana* propagation and phytochemical production.

## Introduction

Pokeweed (*Phytolacca americana* L.) is a perennial herbaceous species belonging to the family Phytolaccaceae and is native to North America. The species has been introduced to several regions of Asia and Europe and is recognized for its rapid growth and ecological adaptability (Trunjaruen, Luecha & Taratima, 2022). *P. americana* has a long history of ethnomedicinal use for the treatment of conditions including rheumatism, arthritis, dermatitis, and infectious diseases (Ryan, 2014). Phytochemical studies have shown that *P. americana* produces diverse secondary metabolites, including phenolic compounds, flavonoids, triterpenoid saponins, anthocyanins, and ribosome-inactivating proteins such as pokeweed antiviral protein (PAP), which have been associated with various biological activities (Marinas et al., 2021). Despite this phytochemical potential, the production and utilization of pokeweed-derived bioactive compounds remain limited by variation in metabolite accumulation among plant sources, environmental effects on phytochemical profiles, and concerns associated with harvesting plant material from natural populations. Therefore, controlled and sustainable systems for the propagation and production of *P. americana* biomass are of considerable interest.

Plant tissue culture provides a controlled approach for the rapid multiplication of plant material under defined environmental and nutritional conditions. In medicinal plants, *in vitro* culture can also provide a more consistent source of plant biomass and phytochemicals than field cultivation, where environmental conditions may influence metabolite accumulation (Efferth & Greten, 2012). However, conventional semi-solid culture systems are relatively labor-intensive and difficult to scale up, whereas continuous liquid cultures may improve nutrient availability but can result in excessive hydration and abnormal tissue development (George & Manuel, 2013; De Carlo et al., 2021). Temporary immersion systems (TIS) provide an alternative approach by periodically exposing plant tissues to liquid medium while allowing intervening aeration periods. This intermittent contact can improve nutrient availability and gas exchange while reducing the physiological problems associated with continuous immersion (Persson, 2012; García-Ramírez, 2023). TIS has therefore been increasingly applied for the large-scale propagation of horticultural and medicinal plants, with advantages in automation, biomass production, and process scalability (Watt, 2012; Murthy et al., 2023; Palladino et al., 2024).

The physical culture environment and immersion regime can influence not only shoot growth and biomass accumulation but also the accumulation of plant secondary metabolites. Previous studies have demonstrated that bioreactor-based culture systems can enhance metabolite production in several medicinal plants, including *Taxus* spp., *Withania somnifera*, and *Bacopa monnieri* (Yue et al., 2016; Sharma et al., 2019; Ahmad et al., 2024). These findings suggest that evaluation of phytochemical characteristics alongside growth responses is important when developing a culture system intended for both plant propagation and production of phytochemical-rich biomass. However, the responses of *P. americana* nodal explants to different culture systems and TIS immersion regimes have not been sufficiently characterized. Therefore, this study aimed to compare solid, liquid, and TIS-based culture systems for the propagation of *P. americana* using nodal explants containing axillary buds. Different TIS immersion durations and frequencies were evaluated for their effects on shoot development, leaf growth, callus formation, and biomass accumulation. In addition, total phenolic content (TPC), total flavonoid content (TFC), and 2,2-diphenyl-1-picrylhydrazyl (DPPH) radical-scavenging activity were assessed to determine how the culture conditions influenced the phytochemical characteristics of the resulting plant material. The combined evaluation of morphogenic and phytochemical responses was intended to identify suitable culture conditions for the controlled propagation and production of phytochemical-rich *P. americana* biomass.

## Materials & Methods

### Plant materials

Mature, fully ripened fruits of *Phytolacca americana* L., characterized by dark purple coloration and a soft texture, were collected from cultivated plants in Sakon Nakhon Province, Thailand (17°27′24.1″N, 103°27′20.2″E). The species was authenticated by Dr. Sakuntala Ninkaew, and a voucher specimen was deposited at the KKU Herbarium (KKU No. 26576). Seeds were manually removed from the berries and subjected to surface sterilization following the procedure described by Trunjaruen, Luecha & Taratima (2022). Briefly, the seeds were immersed in concentrated sulfuric acid (H_2_SO_4_) for 15 min, followed by thorough rinsing under running tap water. The seeds were then shaken in 30% (v/v) Clorox solution for 30 min and subsequently rinsed three times with sterile distilled water, with each rinse lasting at least 5 min. For seed germination, the sterilized seeds were cultured on Murashige and Skoog (MS) basal medium (Murashige & Skoog, 1962) supplemented with 30 g/L sucrose. The medium pH was adjusted to 5.8 before adding eight g/L agar and was subsequently sterilized by autoclaving at 121 °C for 20 min. Cultures were incubated at 25 ± 2 °C under a 16 h light/8 h dark photoperiod with a light intensity of 40 µmol m^−2^ s^−1^ for six weeks. The resulting seedlings were subsequently used as the source material for the experiments.

### Culture systems for axillary shoot proliferation from nodal explants

One-centimeter nodal explants containing pre-existing axillary buds were excised from six-week-old *P. americana* seedlings and used as the initial explants for shoot proliferation. Three culture systems were evaluated: solid culture, shaken liquid culture, and a TIS. The TIS bioreactor consisted of two interconnected vessels, one containing the plant material and the other containing the liquid culture medium. The vessels were connected by a silicone tube, and sterile air was supplied through 0.22-µm pore-size filters fitted to the air inlet and outlet. A timer-controlled system regulated the immersion of the explants in the liquid medium according to the immersion schedules shown in Table 1.

**Table 1:** Treatment design for the temporary immersion system (TIS) bioreactor based on immersion durations and daily frequency.

**Immersion durations**	**Times per day**		
	**1**	**2**	**3**
5 min	T1	T2	T3
10 min	T4	T5	T6

For all three culture systems, the culture medium consisted of MS basal medium supplemented with two mg/L kinetin. For solid culture, 0.8% (w/v) agar was added as the gelling agent, whereas the liquid culture and TIS were maintained without agar. For the solid and shaken liquid cultures, 20 mL of culture medium was dispensed into 4-oz glass vessels. The TIS was supplied with 200 mL of liquid medium. Shaken liquid cultures were maintained on a rotary shaker at 100 rpm. The explant density was standardized at 50 explants per liter of culture medium across all culture systems. The culture medium was adjusted to pH 5.8 before autoclaving at 121 °C for 15 min. All cultures were maintained at 25 ± 2 °C under a 16 h light/8 h dark photoperiod with a light intensity of 40 µmol m^−2^ s^−1^ for six weeks.

The TIS treatments were established using different immersion durations and frequencies, as detailed in Table 1. During each immersion cycle, the liquid medium was transferred to the cultivation vessel to immerse the explants and was subsequently returned to the medium reservoir. Growth responses were evaluated based on survival percentage, response percentage, number of shoots per explant, shoot length, number of leaves per explant, leaf width, leaf length, callus formation percentage, and callus size. Fresh and dry biomass were determined from the entire cultured material, including shoots and any callus tissue attached to the basal region of the explants. Phytochemical contents and antioxidant activity were subsequently evaluated using the same cultured material.

### Sample extraction

The entire cultured material, consisting of shoots with attached basal callus when present, was collected from each replicate and used for phytochemical extraction. Samples were dried at 45 °C for five days and finely powdered prior to extraction. For each replicate, 100 mg of powdered material was randomly selected and suspended in one mL of methanol. The samples were sonicated at 30 °C for 30 min, followed by centrifugation at 14,000 rpm for 5 min. The extraction procedure was repeated for five successive cycles using fresh methanol for each cycle, and the supernatants were pooled. The solvent was subsequently removed using a vacuum oven at 25 °C for 48 h. The resulting extracts were used to determine TPC, TFC, and DPPH radical-scavenging activity.

### Total phenolic content

TPC was determined using the Folin–Ciocalteu colorimetric method according to Hmamou et al. (2022). Briefly, 20 μL of methanolic extract (one mg/mL) was mixed with 100 μL of 10% (v/v) Folin–Ciocalteu reagent and 80 μL of 7% (w/v) sodium carbonate (Na_2_CO_3_) in a 96-well microplate. The reaction mixture was incubated at room temperature in the dark for 30 min, and the absorbance was measured at 760 nm. A calibration curve was prepared using gallic acid at concentrations ranging from 5 to 640 μg/mL. The calibration equation was *y* = 0.0053*x* + 0.0866, with a coefficient of determination (R^2^) of 0.9979. TPC was expressed as milligrams of gallic acid equivalents per gram of extract (mg GAE/g extract).

### Total flavonoid content

TFC was determined using the aluminum chloride colorimetric method according to Barbouchi, Elamrani & El Idrissi (2020). Briefly, 10 μL of methanolic extract (one mg/mL) was mixed with six μL of 5% (w/v) sodium nitrite (NaNO_2_) and incubated for 5 min. Subsequently, 12 μL of 10% (w/v) aluminum chloride (AlCl_3_) was added, followed by incubation for 5 min. Then, 60 μL of 1 M sodium hydroxide (NaOH) and 112 μL of deionized water were added. The absorbance was measured at 510 nm. A calibration curve was prepared using quercetin at concentrations ranging from 10 to 640 μg/mL. The calibration equation was *y* = 0.0001*x* + 0.0004, with a coefficient of determination (R^2^) of 0.9964. TFC was expressed as milligrams of quercetin equivalents per gram of extract (mg QE/g extract).

### Antioxidant activity

The antioxidant activity of the extracts was determined using the DPPH radical-scavenging assay according to Li et al. (2021), with minor modifications. Equal volumes (100 μL) of methanolic extract at different concentrations and DPPH solution were mixed and incubated in the dark for 30 min. The absorbance was subsequently measured at 517 nm. Ascorbic acid was used as the positive control, and methanol was used as the blank. The percentage of free radical scavenging activity (FRSA) was calculated as follows:

\begin{eqnarray*}\mathrm{FRSA}~(\%)=[1-(\mathrm{A_sample}/\mathrm{A_control})]\times 100. \end{eqnarray*}

The IC_50_ value, defined as the extract concentration required to reduce the initial DPPH radical concentration by 50%, was determined from the concentration–response curve. Lower IC_50_ values indicate stronger DPPH radical-scavenging activity.

### Statistical analysis

All experiments were conducted using five independent replicates, and the results were expressed as mean ± standard error (SE). Data were analyzed using analysis of variance (ANOVA), and significant differences among treatment means were determined using Duncan’s multiple range test at *p* < 0.05 with SPSS software (IBM Corp., Armonk, NY, USA). Multivariate analyses, including partial least squares discriminant analysis (PLS-DA), variable importance in projection (VIP) scoring, and hierarchical clustering with heatmap visualization, were performed using MetaboAnalyst version 5.0 to explore patterns of variation among culture systems and identify variables contributing to treatment discrimination.

## Results

### Growth and morphological responses

Nodal explants of *P. americana* showed 100% survival and response rates across all culture systems and TIS treatments (Table 2). Despite the comparable survival and response rates, marked differences were observed in subsequent shoot and leaf development. The shaken liquid culture resulted in the lowest overall growth performance, with significantly lower shoot number, shoot length, leaf number, leaf width, and leaf length than several solid and TIS treatments. In contrast, the TIS treatments generally supported greater shoot and leaf development.

**Table 2:** Effect of culture media and bioreactor immersion conditions on shoot and leaf development from nodal explants of pokeweed.

**Treatments**	**Survival rate (%)**	**Response rate (%)**	**No. of shoot/ explant**	**Shoot length (cm)**	**No. of leaf/ explant**	**Leaf width (cm)**	**Leaf length (cm)**
Solid culture	100.00 ± 0.00^a^	100.00 ± 0.00^a^	2.80 ± 0.58^ab^	3.62 ± 0.29^cd^	13.40 ± 1.75^a^	0.96 ± 0.06^a^	2.62 ± 0.38^b^
Liquid culture	100.00 ± 0.00^a^	100.00 ± 0.00^a^	1.60 ± 0.24^b^	2.46 ± 0.22^d^	6.00 ± 0.32^c^	0.32 ± 0.04^d^	1.44 ± 0.15^c^
T1	100.00 ± 0.00^a^	100.00 ± 0.00^a^	2.47 ± 0.13^ab^	4.01 ± 0.39^cd^	8.93 ± 0.94^bc^	0.42 ± 0.00^de^	2.44 ± 0.05^bc^
T2	100.00 ± 0.00^a^	100.00 ± 0.00^a^	2.13 ± 0.13^ab^	3.79 ± 0.19^cd^	10.80 ± 0.53^ab^	0.36 ± 0.02^de^	2.21 ± 0.25^bc^
T3	100.00 ± 0.00^a^	100.00 ± 0.00^a^	2.47 ± 0.07^ab^	4.41 ± 0.64^a−c^	8.53 ± 0.47^bc^	0.78 ± 0.03^b^	3.73 ± 0.07^a^
T4	100.00 ± 0.00^a^	100.00 ± 0.00^a^	3.53 ± 0.77^a^	5.04 ± 0.73^a−c^	8.93 ± 2.10^bc^	0.61 ± 0.08^c^	3.75 ± 0.62^a^
T5	100.00 ± 0.00^a^	100.00 ± 0.00^a^	2.60 ± 0.20^ab^	5.43 ± 0.72^ab^	6.93 ± 0.18^bc^	0.60 ± 0.07^c^	4.25 ± 0.39^a^
T6	100.00 ± 0.00^a^	100.00 ± 0.00^a^	3.40 ± 0.31^a^	5.69 ± 0.83^a^	6.87 ± 0.52^bc^	0.52 ± 0.03^cd^	4.79 ± 0.55^a^

**Notes.**

Values are expressed as mean ± SE. Different letters in column indicate significant differences at *p* < 0.05 (Duncan’s multiple range test).

The highest number of shoots per explant was observed in T4 (3.53 ± 0.77) and T6 (3.40 ± 0.31), although these values were not significantly different from several other TIS and solid culture treatments. Shoot length was greatest in T6 (5.69 ± 0.83 cm), followed by T5 (5.43 ± 0.72 cm), whereas the shortest shoots were observed in the shaken liquid culture (2.46 ± 0.22 cm). The greatest leaf width was recorded in the solid culture (0.96 ± 0.06 cm), while among the TIS treatments, T3 produced the widest leaves (0.78 ± 0.03 cm). Leaf length was greatest in T6 (4.79 ± 0.55 cm), although this value was not significantly different from T3, T4, and T5. Overall, the results indicate that TIS, particularly treatments with longer immersion durations, supported shoot elongation and leaf expansion, whereas continuous shaken liquid culture resulted in comparatively poor shoot and leaf development (Table 2). Representative morphological responses were shown in Fig. 1.

**Figure 1:**
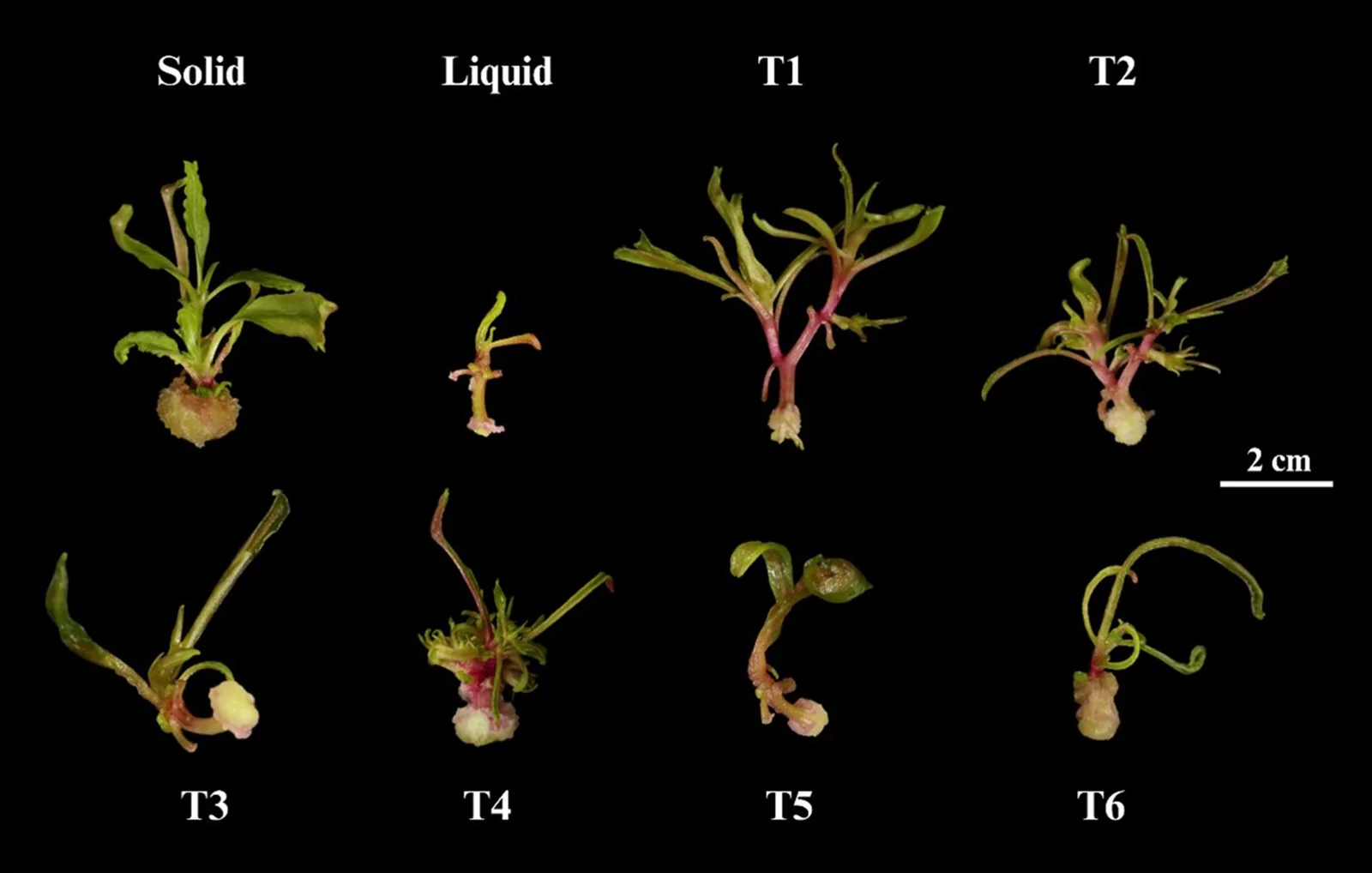
Morphological features. Morphological responses of *P. americana* nodal explants cultured for 6 weeks in MS medium supplemented with 2 mg/L kinetin under different culture systems: solid medium, liquid medium, and temporary immersion bioreactor systems (T1–T6).

### Basal callus formation as a secondary response

Although the experiment was designed to evaluate axillary shoot proliferation from nodal explants, callus formation was also observed at the basal region of the explants under all culture systems (Fig. 1, Table 3). The observed callus formation percentages ranged from 60.00 ± 24.49% in the shaken liquid culture to 100.00 ± 0.00% in the solid culture and T3–T6 treatments; however, these differences were not statistically significant. In contrast, significant differences were observed in callus width. The solid culture produced the widest callus (1.38 ± 0.09 cm), whereas the smallest callus was observed in the shaken liquid culture (0.40 ± 0.17 cm).

**Table 3:** Effects of culture media and bioreactor immersion conditions on callus formation and phytochemical contents of pokeweed.

**Treatments**	**Callus formation (%)**	**Callus width (cm)**	**Fresh biomass (g)**	**Dry biomass (g)**	**TPC (mg GAE/ g extract)**	**TFC (mg QE/g extract)**	**IC**_**50**_ **by DPPH (µg/mL)**
Solid culture	100.00 ± 0.00^a^	1.38 ± 0.09^a^	1.14 ± 0.10^ab^	0.07 ± 0.01^b^	6.76 ± 0.19^e^	31.25 ± 0.94^a^	1046.21 ± 14.92^cd^
Liquid culture	60.00 ± 24.49^a^	0.40 ± 0.17^c^	0.46 ± 0.08^b^	0.04 ± 0.01^b^	11.72 ± 0.41^c^	16.25 ± 1.56^e^	619.27 ± 3.96^f^
T1	86.67 ± 6.67^a^	0.86 ± 0.14^b^	1.28 ± 0.08^ab^	0.06 ± 0.00^b^	6.19 ± 0.19^e^	20.78 ± 0.60^c^	553.25 ± 16.46^g^
T2	73.33 ± 13.33^a^	0.80 ± 0.13^b^	1.35 ± 0.11^ab^	0.06 ± 0.00^b^	6.51 ± 0.03^e^	16.61 ± 0.60^de^	1245.01 ± 4.50^a^
T3	100.00 ± 0.00^a^	1.08 ± 0.01^ab^	1.59 ± 0.12^ab^	0.07 ± 0.01^b^	9.93 ± 0.25^d^	24.67 ± 0.87^b^	746.17 ± 5.90^e^
T4	100.00 ± 0.00^a^	1.13 ± 0.06^ab^	1.64 ± 0.56^ab^	0.10 ± 0.02^ab^	13.37 ± 0.34^b^	26.75 ± 0.42^b^	1161.55 ± 19.89^b^
T5	100.00 ± 0.00^a^	0.95 ± 0.02^b^	1.80 ± 0.95^a^	0.07 ± 0.04^ab^	14.82 ± 0.10^a^	26.89 ± 0.91^b^	1033.50 ± 18.45^d^
T6	100.00 ± 0.00^a^	1.11 ± 0.07^ab^	2.07 ± 0.50^a^	0.12 ± 0.03^a^	11.18 ± 0.06^c^	19.81 ± 0.61^cd^	1084.10 ± 7.47^c^

**Notes.**

Values are expressed as mean ± SE. Different letters in column indicate significant differences at *p* < 0.05 (Duncan’s multiple range test). Biomass = shoots together with attached basal callus tissue.

The biomass of the cultured material, comprising shoots and any callus tissue attached to the basal region of the explants, also differed among treatments. The highest fresh biomass was observed in T5 (1.80 ± 0.95 g) and T6 (2.07 ± 0.50 g), whereas the shaken liquid culture produced the lowest fresh biomass (0.46 ± 0.08 g). Dry biomass was highest in T6 (0.12 ± 0.03 g), while the lowest values were observed in the solid and shaken liquid cultures and T1–T3. These results indicate that TIS treatments, particularly T5 and T6, supported greater accumulation of total cultured biomass than the shaken liquid culture.

### Phytochemical content and antioxidant activity

TPC varied significantly among the culture systems and TIS treatments, ranging from 6.19 to 14.82 mg gallic acid equivalents per gram (GAE/g) extract (Table 3). The highest TPC was observed in T5 (14.82 ± 0.10 mg GAE/g extract), followed by T4 (13.37 ± 0.34 mg GAE/g extract) and T6 (11.18 ± 0.06 mg GAE/g extract). The lowest TPC values were recorded in T1 (6.19 ± 0.19 mg GAE/g extract) and the solid culture (6.76 ± 0.19 mg GAE/g extract), which were not significantly different from each other.

TFC ranged from 16.25 to 31.25 mg quercetin equivalents per gram (QE/g) extract. The solid culture produced the highest TFC (31.25 ± 0.94 mg QE/g extract), whereas the shaken liquid culture showed the lowest value (16.25 ± 1.56 mg QE/g extract). Among the TIS treatments, T5 (26.89 ± 0.91 mg QE/g extract) and T4 (26.75 ± 0.42 mg QE/g extract) exhibited relatively high TFC values, followed by T3 (24.67 ± 0.87 mg QE/g extract). T6, T1, and T2 showed lower TFC values of 19.81 ± 0.61, 20.78 ± 0.60, and 16.61 ± 0.60 mg QE/g extract, respectively.

Antioxidant activity, determined by the DPPH radical-scavenging assay and expressed as IC_50_ values, also differed significantly among the culture treatments (Table 3). Lower IC_50_ values indicate stronger radical-scavenging activity. The lowest IC_50_ was observed in T1 (553.25 ± 16.46 µg/mL), followed by the shaken liquid culture (619.27 ± 3.96 µg/mL), whereas T2 exhibited the highest IC_50_ (1,245.01 ± 4.50 µg/mL). These results indicate that the culture systems and TIS immersion regimes affected phenolic content, flavonoid content, and antioxidant activity differently, with no single treatment producing the highest values across all phytochemical parameters.

Because the biomass used for phytochemical analysis consisted of the entire cultured material, including shoots and any callus tissue attached to the basal region of the explants, differences in tissue composition among treatments may have contributed to the observed variation in phytochemical content and antioxidant activity.

### Multivariate analysis

PLS-DA was used to examine the relationships between the measured growth, callus-related, biomass, phytochemical, and antioxidant parameters and the different culture treatments. The first and second latent variables explained 52.8% and 18.1% of the total variance, respectively (Fig. 2). The PLS-DA biplot showed distinct associations between culture systems and specific trait profiles. The solid culture treatment was positioned in the direction of callus width, callus formation, leaf width, and shoot number. In contrast, the TIS treatments were distributed in the direction of shoot length, leaf length, fresh biomass (FW), dry biomass (DW), TPC, and DPPH activity (expressed as IC_50_), although the individual TIS treatments did not form clearly separated clusters. The shaken liquid culture was positioned away from most of the growth-related variables, consistent with its comparatively lower growth and biomass values.

**Figure 2:**
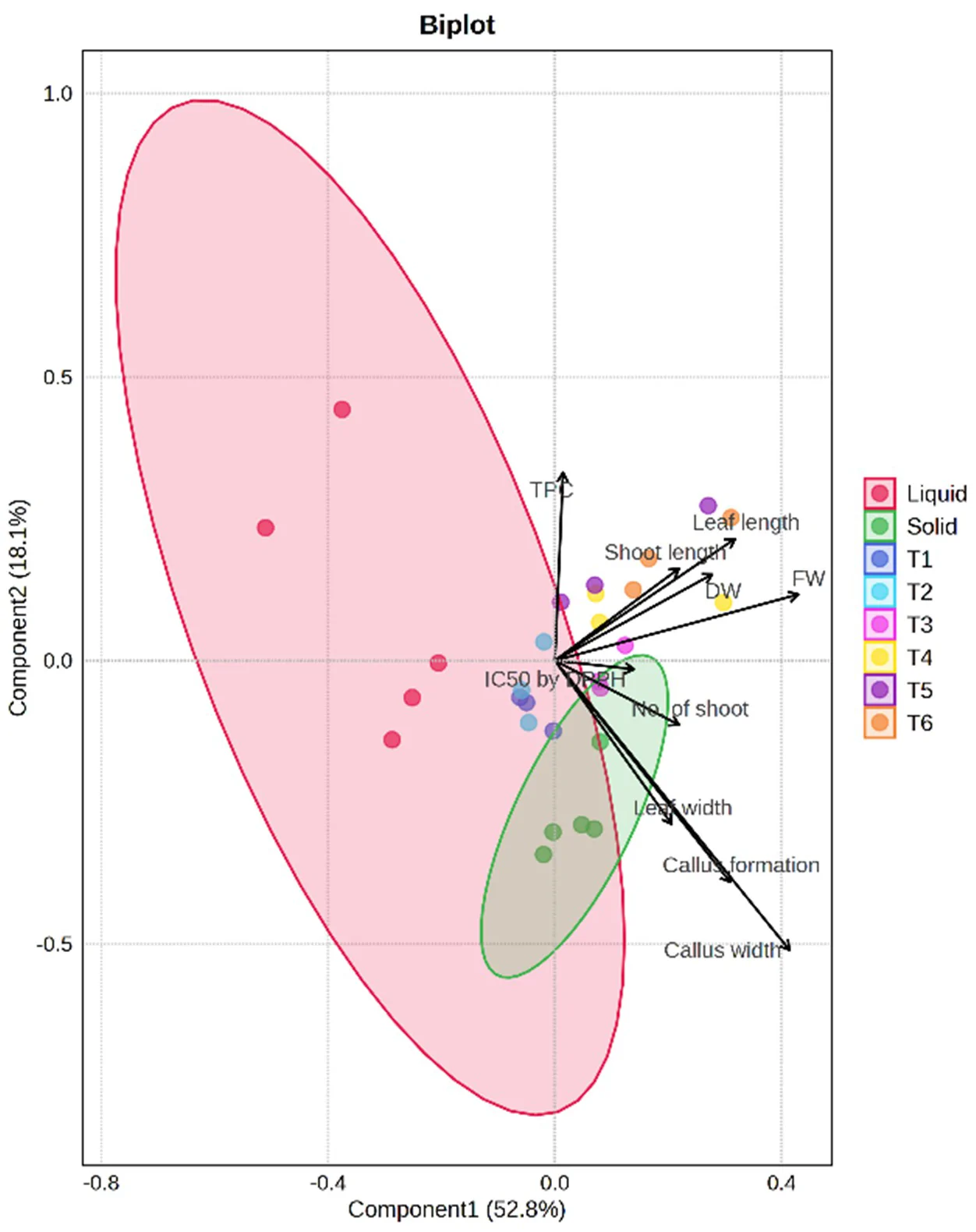
PLS-DA analysis. PLS-DA biplot of *P. americana* nodal explants cultured for 6 weeks in MS medium supplemented with 2 mg/L kinetin under different culture systems: solid medium, liquid medium, and temporary immersion bioreactors treatments (T1–T6).

The VIP analysis identified fresh biomass, leaf length, callus width, shoot length, and dry biomass as the variables contributing most strongly to treatment discrimination (VIP > 1.0) (Fig. 3). Callus formation, shoot number, IC_50_, and TPC showed intermediate contributions, whereas TFC, leaf width, and leaf number contributed relatively less to the separation among culture treatments. These findings indicate that differences in biomass accumulation and shoot development were major contributors to the observed separation of culture systems.

**Figure 3:**
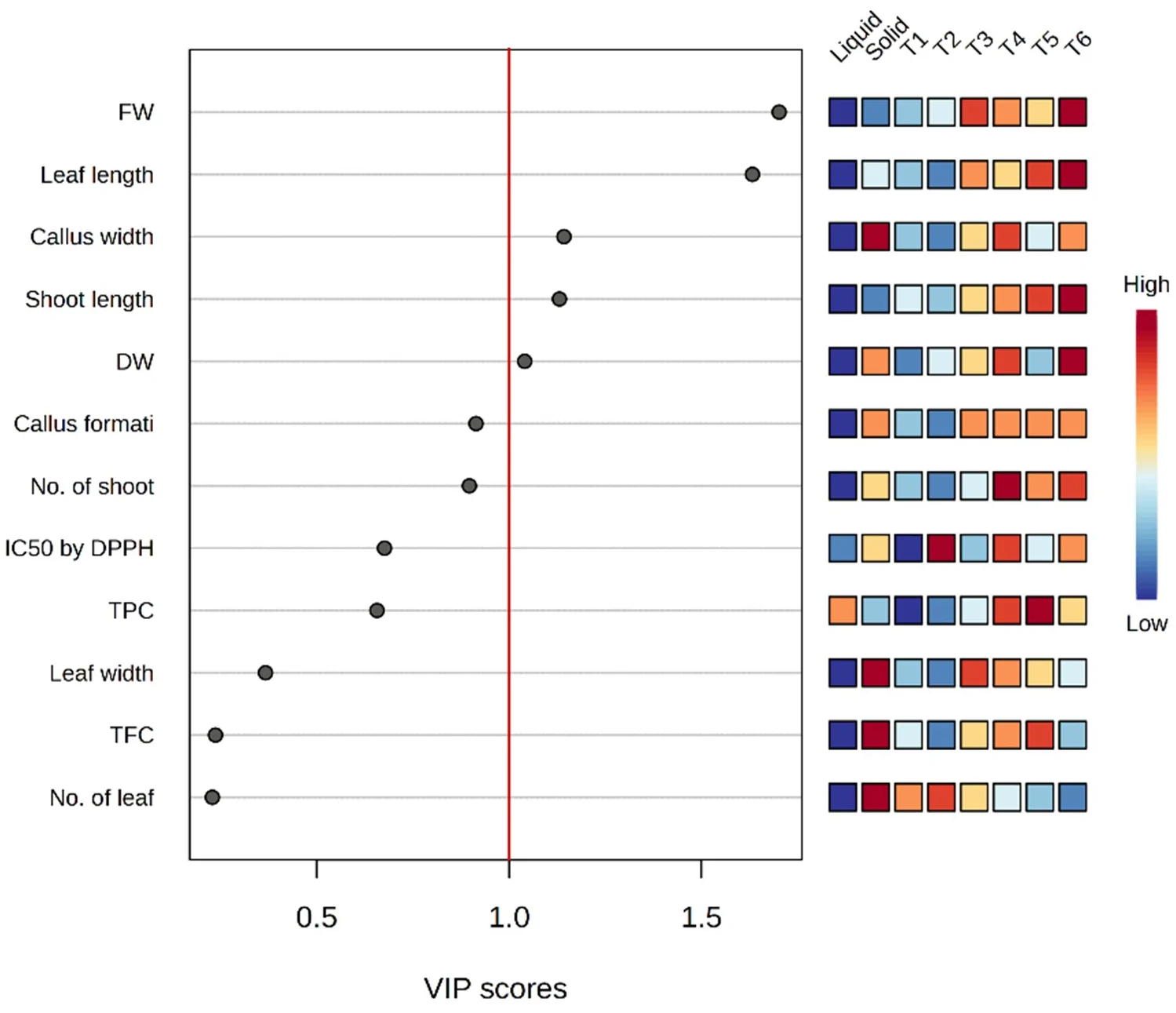
VIP score. VIP scores from PLS-DA of *P. americana* nodal explants cultured for 6 weeks in MS medium supplemented with 2 mg/L kinetin under different culture systems: solid medium, liquid medium, and temporary immersion bioreactor treatments (T1–T6).

The hierarchical clustering heatmap provided a complementary view of the treatment-specific patterns (Fig. 4). The shaken liquid culture was characterized by relatively low values for most growth and biomass-related parameters, although TPC remained comparatively high. Several TIS treatments, particularly T4–T6, showed relatively high values for shoot length, leaf length, fresh biomass, dry biomass, and TPC. The solid culture was characterized by relatively high values for leaf number, shoot number, TFC, DPPH antioxidant activity, and callus-related traits. Overall, the multivariate analyses indicated that the culture systems produced distinct combinations of growth, biomass, callus-related, and phytochemical characteristics rather than a single treatment consistently outperforming the others across all measured parameters.

**Figure 4:**
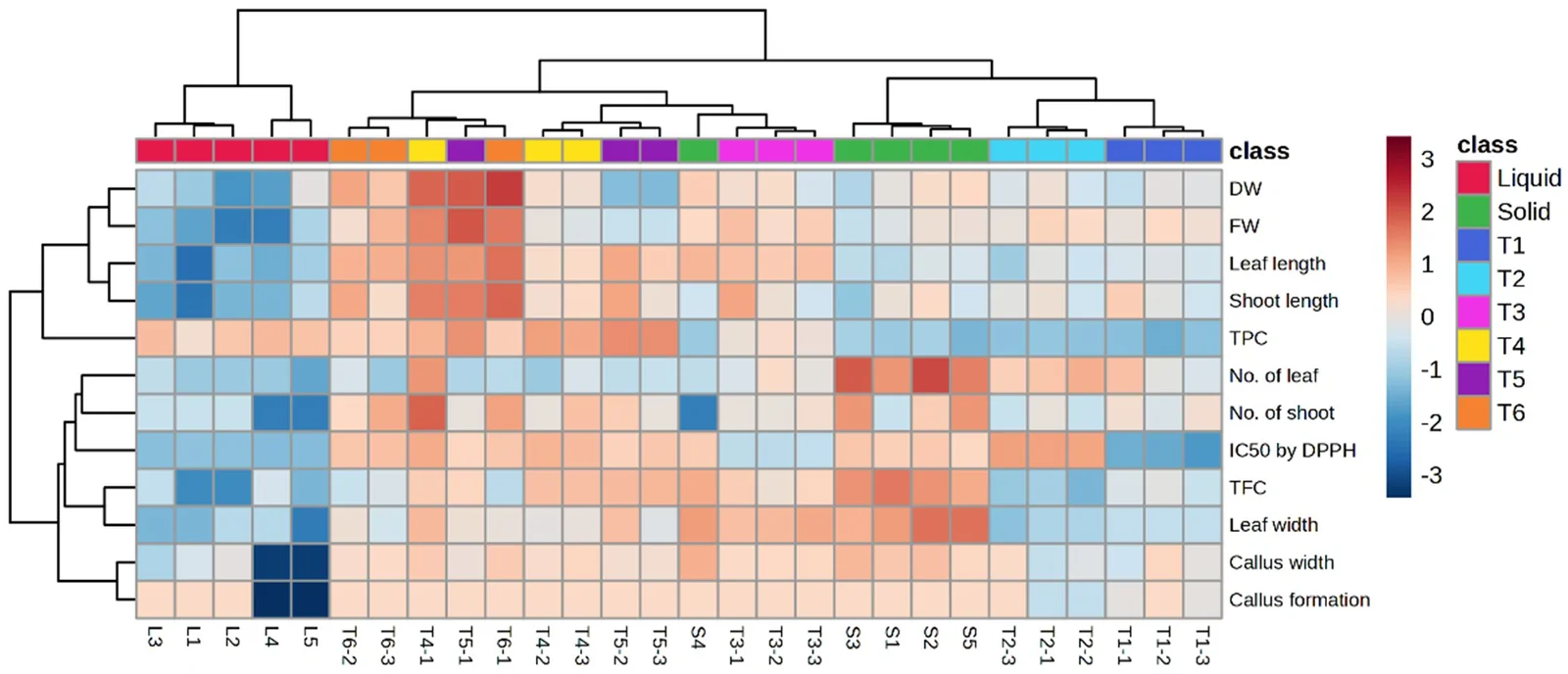
Heatmap analysis. Heatmap analysis of *P. americana* nodal explants cultured for 6 weeks in MS medium supplemented with 2 mg/L kinetin under different culture systems: solid medium, liquid medium, and temporary immersion bioreactor treatments (T1–T6), showing hierarchical clustering of growth and biochemical traits.

## Discussion

### Growth and morphological responses

The present study demonstrates that the culture system and TIS immersion regime influenced the growth and morphological responses of *P. americana* nodal explants. Although all treatments maintained 100% explant survival and response rates, subsequent shoot and leaf development differed among culture conditions. The shaken liquid culture consistently resulted in lower shoot and leaf growth, whereas several TIS treatments, particularly T4–T6, supported greater shoot elongation and shoot production. T6 produced the longest shoots and leaves and the highest dry biomass, while T4 and T6 produced the greatest numbers of shoots per explant. These findings are consistent with previous reports showing that temporary immersion can improve plant growth and shoot multiplication by providing intermittent access to liquid nutrients while reducing the duration of complete submergence compared with continuous liquid culture (Makowski et al., 2023; Uma et al., 2021).

The relatively poor growth observed under shaken liquid culture may be associated with the continuous exposure of explants to liquid medium. Continuous immersion can alter gas exchange and the physical environment surrounding plant tissues, potentially affecting oxygen availability and nutrient uptake. Such conditions have been reported to influence growth and morphogenic responses in *in vitro* cultures (Mirzabe et al., 2022). However, these mechanisms were not directly examined in the present study and therefore should be considered possible explanations rather than demonstrated physiological responses. The superior growth observed under several TIS regimes may instead reflect the intermittent exposure of explants to the liquid medium, allowing repeated nutrient uptake while providing periods of exposure to the gaseous phase between immersion cycles.

Callus formation occurred as a secondary response at the basal region of the nodal explants under all culture systems, although the frequency of callus formation did not differ significantly among treatments. In contrast, callus width varied significantly, with the largest callus observed under solid culture and the smallest under shaken liquid culture. The greater callus development observed on solid medium may be related to the physical support and relatively stable water availability provided by the solid medium. Similar effects of the physical properties of the culture matrix on callus growth and tissue development have been reported in plant tissue culture systems (Pasternak & Steinmacher, 2024). The smaller callus observed in shaken liquid culture may reflect differences in tissue–medium interactions and the continuous movement of the explants; however, the physiological basis of this response was not directly investigated in this study.

The TIS treatments also affected biomass accumulation. T5 and T6 produced the highest fresh biomass values, although these values were not significantly different from several other treatments, whereas T6 produced the highest dry biomass. Because biomass was determined from the entire cultured material, including shoots and any callus tissue attached to the basal region of the explants, the observed differences represent changes in total cultured biomass rather than callus-specific biomass. The greater biomass observed under some TIS regimes may be associated with the repeated exposure of explants to nutrient medium and the alternating liquid and gaseous phases characteristic of temporary immersion systems. Previous studies have suggested that immersion frequency and duration can influence nutrient availability, gas exchange, and biomass accumulation in TIS-based cultures (De Carlo et al., 2021; Makowski et al., 2024). Nevertheless, these physiological mechanisms were not directly measured in the present study and should therefore be interpreted as possible explanations. Overall, the results indicate that TIS provided favorable conditions for shoot growth and biomass accumulation under several immersion regimes, while the responses of individual growth traits varied according to the immersion program. Importantly, no single culture treatment was superior across all measured growth and morphological parameters. These findings suggest that the selection of an appropriate TIS regime for *P. americana* should depend on the desired production objective, such as shoot multiplication, shoot elongation, or biomass accumulation.

### Phytochemical characteristics and antioxidant activity

The culture system and TIS immersion regime also influenced the phytochemical characteristics of *P. americana* explants. TPC was highest in T5 (10 min immersion, twice per day), whereas TFC was highest in the solid culture system. These differences indicate that the culture environment influenced phenolic and flavonoid accumulation differently rather than producing a uniform increase in all phytochemical groups. Phenolic compounds are important components of plant secondary metabolism and contribute to antioxidant protection and adaptation to environmental stress, while flavonoids have diverse physiological functions associated with plant growth and development (Manzoor et al., 2023; Zagoskina et al., 2023). In plant tissue culture, changes in the culture environment can alter secondary metabolism and promote differential accumulation of bioactive compounds (Ozyigit et al., 2023). Therefore, the distinct phytochemical profiles observed among culture systems may reflect differences in the physiological conditions experienced by the explants. However, these proposed relationships were not directly investigated in the present study and should be considered possible interpretations rather than demonstrated mechanisms.

Antioxidant activity also varied among culture conditions and did not correspond directly to TPC or TFC. The strongest DPPH radical-scavenging activity, indicated by the lowest IC_50_ value, was observed in T1, whereas T2 showed the weakest activity. Interestingly, T6 produced the highest dry biomass but did not show the highest TPC, TFC, or antioxidant activity. This indicates that biomass accumulation and phytochemical accumulation were not necessarily enhanced under the same culture conditions. Such differences may reflect variation in the allocation of resources between growth and secondary metabolism in response to the culture microenvironment. In TIS-based cultures, immersion frequency and duration can influence nutrient availability, aeration, hydration status, and culture-related stress, thereby affecting both biomass production and secondary metabolism (Mirzabe et al., 2022; Ozyigit et al., 2023). Nevertheless, these factors were not directly measured in this study and should not be interpreted as definitive mechanisms.

It should also be noted that the phytochemical analyses were performed using the same plant material used for biomass determination, consisting of whole plantlets with any callus tissue remaining attached at the basal region. Because the relative contribution of callus and shoot tissues may have differed among treatments, differences in phytochemical composition may partly reflect differences in tissue composition. Therefore, the present results demonstrate treatment-dependent differences in the overall phytochemical characteristics of the cultured material, but they do not establish a direct causal relationship between phenolic or flavonoid accumulation and shoot propagation. Further studies using separated tissues, time-course sampling, and targeted analysis of individual bioactive compounds would help clarify the relationship between tissue development and secondary metabolism in *P. americana*.

### Multivariate analysis

The combined use of PLS-DA, VIP analysis, and hierarchical clustering provided a complementary view of the relationships between culture treatments and the measured growth, biomass, callus-related, phytochemical, and antioxidant traits. The PLS-DA analysis showed that the major sources of variation among treatments were associated primarily with biomass accumulation and shoot development. Fresh biomass, leaf length, callus width, shoot length, and dry biomass had the highest VIP scores, indicating that these variables contributed strongly to the separation of the culture treatments. In contrast, TFC, leaf width, and leaf number contributed less to treatment discrimination. The hierarchical clustering analysis further supported the presence of treatment-specific trait profiles, with the shaken liquid culture generally characterized by lower growth and biomass values, whereas several TIS treatments, particularly T4–T6, were associated with higher shoot- and biomass-related values. The solid culture showed relatively high values for leaf number, shoot number, TFC, and callus-related traits.

The multivariate results should nevertheless be interpreted with caution. PLS-DA is a supervised classification method and can be susceptible to overfitting, particularly when the number of variables is relatively large compared with the number of independent samples (Rodríguez-Pérez, Fernández & Marco, 2018; Eicher et al., 2020). In the present study, PLS-DA was used primarily as an exploratory approach to identify variables contributing to treatment separation, and independent validation or permutation testing was not performed. Therefore, the observed separation should be considered indicative of treatment-associated patterns rather than definitive classification performance. Future studies incorporating independent experimental batches and additional validation procedures would help confirm the stability of the identified discriminating variables (Ghosh et al., 2020; Mészáros et al., 2025).

The hierarchical clustering and heatmap analyses provided additional support for the treatment-specific patterns but, like PLS-DA, describe associations rather than causal relationships. The distinct responses observed among solid, shaken liquid, and TIS cultures may be related to differences in nutrient availability, tissue–medium interactions, gas exchange, physical support, and immersion dynamics. Previous studies have shown that the physical state of the culture medium can influence nutrient and hormone movement, tissue hydration, gas exchange, and morphogenic responses in plant tissue culture systems (Pandey, Shukla & Misra, 2018; Shukla et al., 2019; Muzika et al., 2024; Nirmal, Teraiya & Joshi, 2023; Ozyigit et al., 2023). However, these factors were not directly measured in the present study and therefore cannot be identified as the underlying mechanisms responsible for the observed responses. Focused physiological and molecular studies would be required to determine the relative contributions of these factors to the responses observed in *P. americana*.

Interestingly, DPPH antioxidant activity contributed less strongly to treatment discrimination than several growth and biomass-related variables. This suggests that antioxidant activity did not closely follow the major morphological and biomass patterns identified by the multivariate analysis. The different responses of growth and phytochemical traits may reflect the complex regulation of phenolic and flavonoid metabolism during *in vitro* growth, in which metabolite accumulation may not necessarily increase proportionally with biomass production. However, the present study did not directly investigate the metabolic pathways underlying these responses.

Furthermore, the phytochemical analyses were conducted at a single sampling point and were based on TPC, TFC, and DPPH radical-scavenging activity rather than targeted quantification of individual compounds. Consequently, temporal changes in metabolite accumulation and treatment-specific changes in individual bioactive compounds may not have been captured. Future studies incorporating multiple sampling times and targeted metabolite profiling could provide a more detailed understanding of the relationship between culture conditions, shoot development, biomass accumulation, and phytochemical production in *P. americana*.

Collectively, the multivariate analyses support the conclusion that different culture systems and TIS regimes generated distinct combinations of morphological, biomass, phytochemical, and antioxidant characteristics. Rather than identifying a single culture system as superior across all traits, the analysis highlights the trait-specific responses of *P. americana* nodal explants to different culture environments. This integrated perspective supports the use of multivariate analysis as a complementary tool for identifying key traits associated with culture-system performance, while further physiological and metabolite-level investigations are needed to clarify the mechanisms underlying these associations.

## Conclusions

This study demonstrates that culture systems and TIS immersion regimes differentially influenced shoot development, biomass accumulation, callus formation, and phytochemical characteristics of *P. americana* nodal explants. The temporary immersion treatments with 10-min immersions once or three times per day supported greater shoot production, with the three-times-per-day treatment producing the longest shoots and leaves and the highest dry biomass. Phytochemical responses varied among treatments, with the highest TPC observed under 10-min immersions twice per day, whereas TFC was highest in solid culture and DPPH radical-scavenging activity was strongest under 5-min immersions once per day. Thus, no single culture condition was superior across all traits, highlighting the importance of selecting an appropriate immersion regime according to the desired production objective. Because biomass and phytochemical analyses were performed using whole cultured material containing shoots and attached basal callus, tissue-specific contributions could not be distinguished. Future studies should therefore examine temporal and tissue-specific phytochemical changes and targeted metabolite profiles to further clarify the relationship between shoot development, biomass accumulation, and phytochemical production in *P. americana*.

## Supplemental Information

10.7717/peerj.21812/supp-1 Supplemental Information 1 Raw data of growth performance and phytochemical content

## Additional Information and Declarations

### Data Availability

The following information was supplied regarding data availability: The data is available in the Supplementary File.
